# Cell-type-specific modulation of targets and distractors by dopamine D1 receptors in primate prefrontal cortex

**DOI:** 10.1038/ncomms13218

**Published:** 2016-10-27

**Authors:** Simon N. Jacob, Maximilian Stalter, Andreas Nieder

**Affiliations:** 1 Department of Animal Physiology, Institute of Neurobiology, Eberhard-Karls-Universität Tübingen, Auf der Morgenstelle 28, 72076 Tübingen, Germany

## Abstract

The prefrontal cortex (PFC) is crucial for maintaining relevant information in working memory and resisting interference. PFC neurons are strongly regulated by dopamine, but it is unknown whether dopamine receptors are involved in protecting target memories from distracting stimuli. We investigated the prefrontal circuit dynamics and dopaminergic modulation of targets and distractors in monkeys trained to ignore interfering stimuli in a delayed-match-to-numerosity task. We found that dopamine D1 receptors (D1Rs) modulate the recovery of task-relevant information following a distracting stimulus. The direction of modulation is cell-type-specific: in putative pyramidal neurons, D1R inhibition enhances and D1R stimulation attenuates coding of the target stimulus after the interference, while the opposite pattern is observed in putative interneurons. Our results suggest that dopaminergic neuromodulation of PFC circuits regulates mental representations of behaviourally relevant stimuli that compete with task-irrelevant input and could play a central role for cognitive functioning in health and disease.

## Abstract (web summary)

Dopaminergic activity in prefrontal cortex is important for working memory but the neural mechanisms of its action are not known. Jacob and colleagues show that dopamine D1 receptors regulate neural responses to relevant stimuli in a cell-type-specific manner in the presence of distractors.

## Graphical abstract

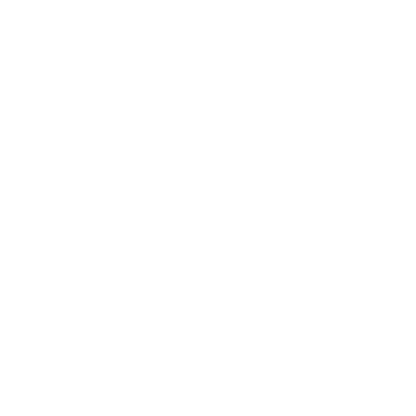

The primate prefrontal cortex (PFC) is a major brain hub for guiding intelligent behaviour^1^,^2^. During cognitive control, stimuli are stored in working memory and processed flexibly from moment to moment depending on the behavioural context and current goals^3^,^4^,^5^,^6^. The PFC can represent task-relevant (target) and distracting stimuli side by side in working memory and select the appropriate information to resist memory interference^7^. However, the neuronal mechanisms that allow PFC circuits to bypass distractors and retrieve target information in such a way are not known.

A large body of experimental evidence has shown that frontal lobe executive functions are strongly regulated by midbrain dopamine neurons^8^,^9^,^10^. Dopaminergic neurons fire phasic bursts of action potentials with short latencies in response to important sensory events^11^,^12^,^13^. Prefrontal dopamine D1 receptors (D1Rs) have been found to modulate the allocation of attention^14^, learning of associations^15^ and rule-based reasoning^16^.

One of the most intensively studied dopamine-dependent PFC functions is spatial working memory^17^,^18^. In rhesus monkeys trained to memorize the location of saccade targets, D1Rs modulate the delay period activity of individual neurons and can improve or disrupt tuning to remembered locations depending on the extent of activation^19^,^20^. Given the increasingly recognized role of D1Rs for executive functions, we hypothesized that dopamine could also be involved in controlling prefrontal processing when multiple events compete for working memory resources—a frequently occurring and more realistic situation than the previously studied presentation of single stimuli. Unregulated dopamine signals are believed to underlie the characteristic inability to filter behaviourally irrelevant information in severe mental disorders^21^,^22^. Dopamine-generated overshooting, aberrant salience represents one of the most influential contemporary theories to explain the cardinal symptoms of psychosis such as sensory hallucinations and intrusions of thought^23^. Experimental data to support any of these concepts are lacking.

To investigate how prefrontal D1Rs modulate working memory for multiple stimuli, we trained two rhesus monkeys to memorize the number of visual items (numerosity) of a target stimulus while resisting other, distracting numerosities^7^. We then recorded single-unit activity from the PFC, while simultaneously applying the D1R antagonist SCH23390 or the D1R agonist SKF38393 to the vicinity of the cells using microiontophoresis^16^,^24^. We considered two possible outcomes. Following directly from current knowledge about dopaminergic modulation of prefrontal working memory^25^, modulation of delay period activity by D1Rs might exclusively affect the encoding of the most recent stimulus, irrespective of its relevance for behaviour. Alternatively, D1Rs could also be involved in modulating the recovery of the originally memorized, task-relevant target information following a distractor. We found evidence for the latter. Our results suggest that dopaminergic neuromodulation of PFC circuits can strengthen mental representations of behaviourally relevant stimuli that compete with task-irrelevant input and could play a central role for cognitive functioning in health and disease.

## Results

### Behavioural task

**Figure 1:**
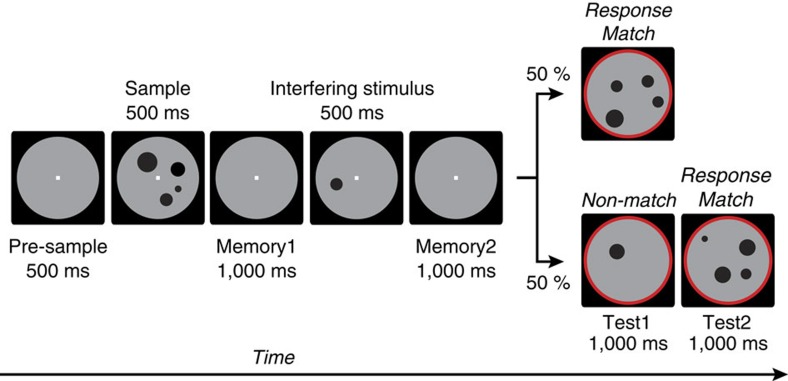
Task protocol. Delayed match-to-sample task. Monkeys had to release a bar if the sample and first test display contained the same number of items (Match) and had to continue holding it if they did not (Non-Match). A task-irrelevant, interfering numerosity presented in the working memory period had to be resisted.

We trained two monkeys on a modified version of a delayed-match-to-numerosity task^7^ with 1, 2 or 4 sample items (Fig. 1). A task-irrelevant interfering numerosity (1, 2 or 4 items) of 500 ms duration was presented during the memory interval in 75% of the trials (25% of the trials each with numerosity 1, 2 or 4). In the remaining 25% of the trials, a blank grey background circle of equal duration replaced the interfering numerosity, that is, no task-irrelevant stimulus was shown (standard delayed-match-to-numerosity task). Low-level visual features of the dot stimuli were controlled to ensure that numerosity was the only relevant stimulus feature^26^,^27^.

We previously reported a detailed analysis of the two monkeys' behavioural performance in an extended version of the distractor task, showing that both animals had learned to respond to the sample numerosity despite memory interference by the task-irrelevant stimulus^7^. In a new set of experiments, we now addressed the role of prefrontal D1R signalling in representing target and interfering information using simultaneous neuronal recordings and microiontophoretic drug application^16^,^24^.

### Iontophoretic drug application and cell-type classification

**Figure 2:**
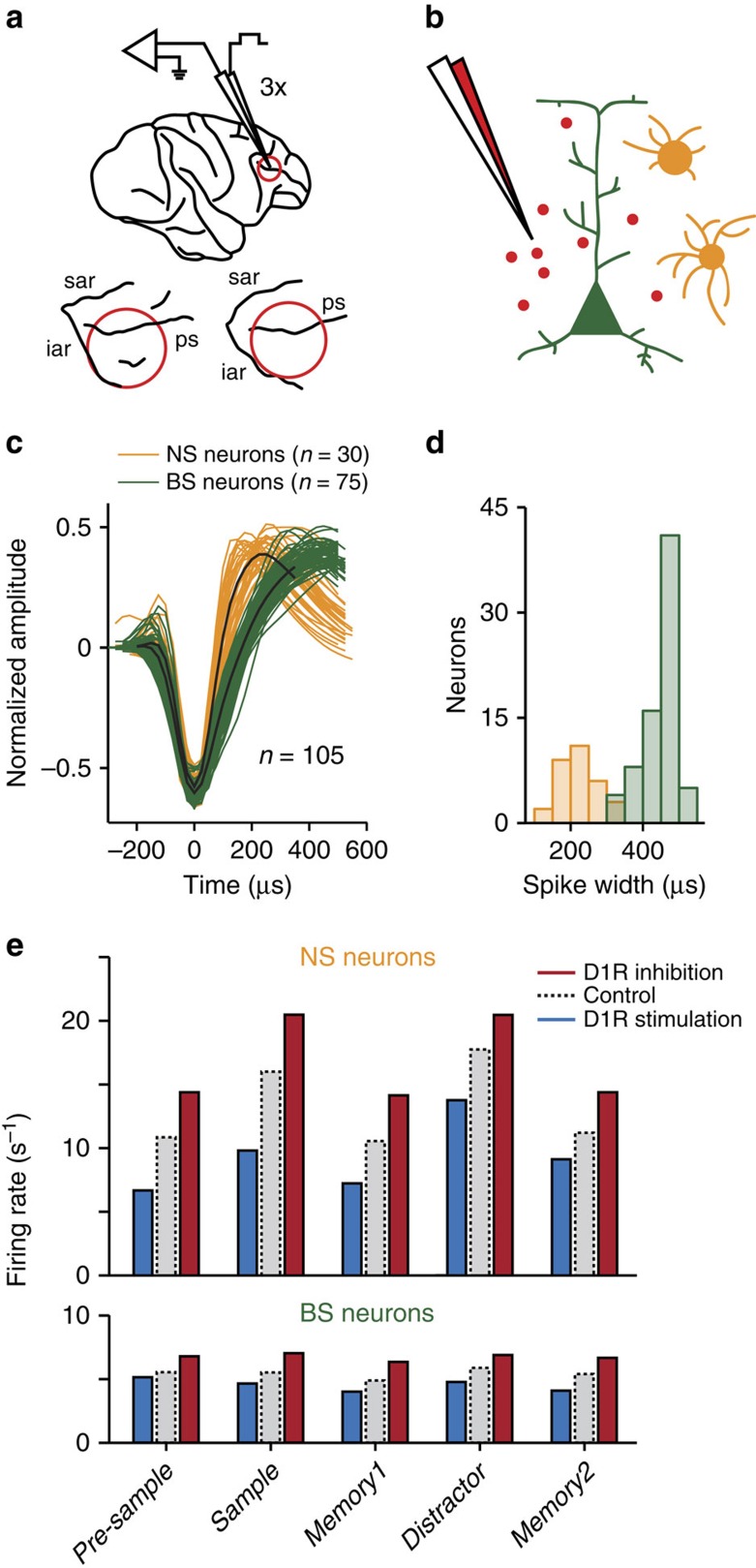
Microiontophoretic drug application and cell-type classification. (**a**) Top: schematic of a rhesus monkey brain depicting the location of simultaneous single-unit recordings and multi-pipette iontophoretic drug application in the lateral PFC. Bottom: anatomical surface reconstruction of the recording sites in monkey R and W (left and right, respectively). (**b**) Schematic of iontophoretic drug application to the local microcircuit consisting of excitatory pyramidal neurons and inhibitory interneurons. (**c**) Spike waveforms for all analysed sample-selective neurons (*P*<0.01 in any task epoch) that were successfully classified as broad-spiking (BS) or narrow-spiking (NS). Cluster centroids for each class are marked in black. (**d**) Distribution of spike widths for the neurons in **c**. (**e**) Firing rates for the neurons in **c** averaged within each task epoch in control conditions, with D1R inhibition (SCH23390) and D1R stimulation (SKF38393).

While the animals performed the task, we iontophoretically applied the selective D1R antagonist SCH23390 (*n*=47 sessions) or the D1R agonist SKF38393 (*n*=54 sessions) through up to three pipettes to the ventral and dorsal bank of the principal sulcus in lateral PFC (Fig. 2a). Control conditions without drug application alternated with drug conditions in each recording session. We recorded from 246 randomly selected single neurons (see Methods; SCH23390: *n*=119; SKF38393: *n*=127; Fig. 2b). A total of 122 neurons (50%; SCH23390: *n*=60; SKF38393: *n*=62) encoded the sample numerosity in at least one trial epoch (three-way analysis of variance (ANOVA) with factors sample (1, 2 or 4), distractor (1, 2 or 4), and iontophoresis condition (control or SCH22390/SKF38393), evaluated at *P*<0.01) and were subjected to further in-depth analysis.

Interneurons and pyramidal cells are thought to play complementary roles in shaping prefrontal cognitive processing, in particular in working memory tasks^28^,^29^. We therefore hypothesized that different cell classes might be associated with distinct response properties after presentation of the target and distractor stimulus. To establish such cell-type specific response profiles, we calculated the average normalized waveform for each individual neuron and used a linear classifier to objectively separate narrow-spiking neurons (NS; putative interneurons) from broad-spiking neurons (BS; putative pyramidal cells). One hundred five neurons (86%; NS: *n*=30; BS: *n*=75) were successfully classified (Fig. 2c). The distribution of the waveforms' peak-to-trough durations was bimodal^30^,^31^ (Fig. 2d). NS neurons had higher firing rates than BS neurons in all trial periods (mean across trial epochs for all trials, NS neurons: 13.5±2.4 spikes per s; BS neurons: 5.5±0.7 spikes per s; *P*=0.001, rank sum test; Fig. 2e). These results suggest that our sample of NS neurons mostly contained inhibitory interneurons, while the BS neurons were mostly pyramidal cells. In both cell classes, firing rates after SCH23390 application were higher than in control trials and lower after SKF38393 application, although this difference did not reach significance (mean across trial epochs, NS neurons (spikes per s): SCH23390 16.0, control 13.3, SKF38393 9.2; *P*=0.25, Kruskal–Wallis test; BS neurons (spikes per s): SCH23390 6.3, control 5.4, SKF38393 4.8; *P*=0.33; Fig. 2e).

### D1R modulation of task-relevant information in PFC

We had previously reported that the neuronal representation of the irrelevant distractor stimulus in PFC is not actively suppressed in this task^7^. Instead, the recovery of target (sample) information in the second memory period preceding the test numerosity is required for correct responses. Thus, we first investigated whether the level of prefrontal D1R activity influenced the neuronal coding strength for the task-relevant sample numerosity at various time points in the trial, focusing in particular on the epoch preceding the monkeys' behavioural choice.

**Figure 3:**
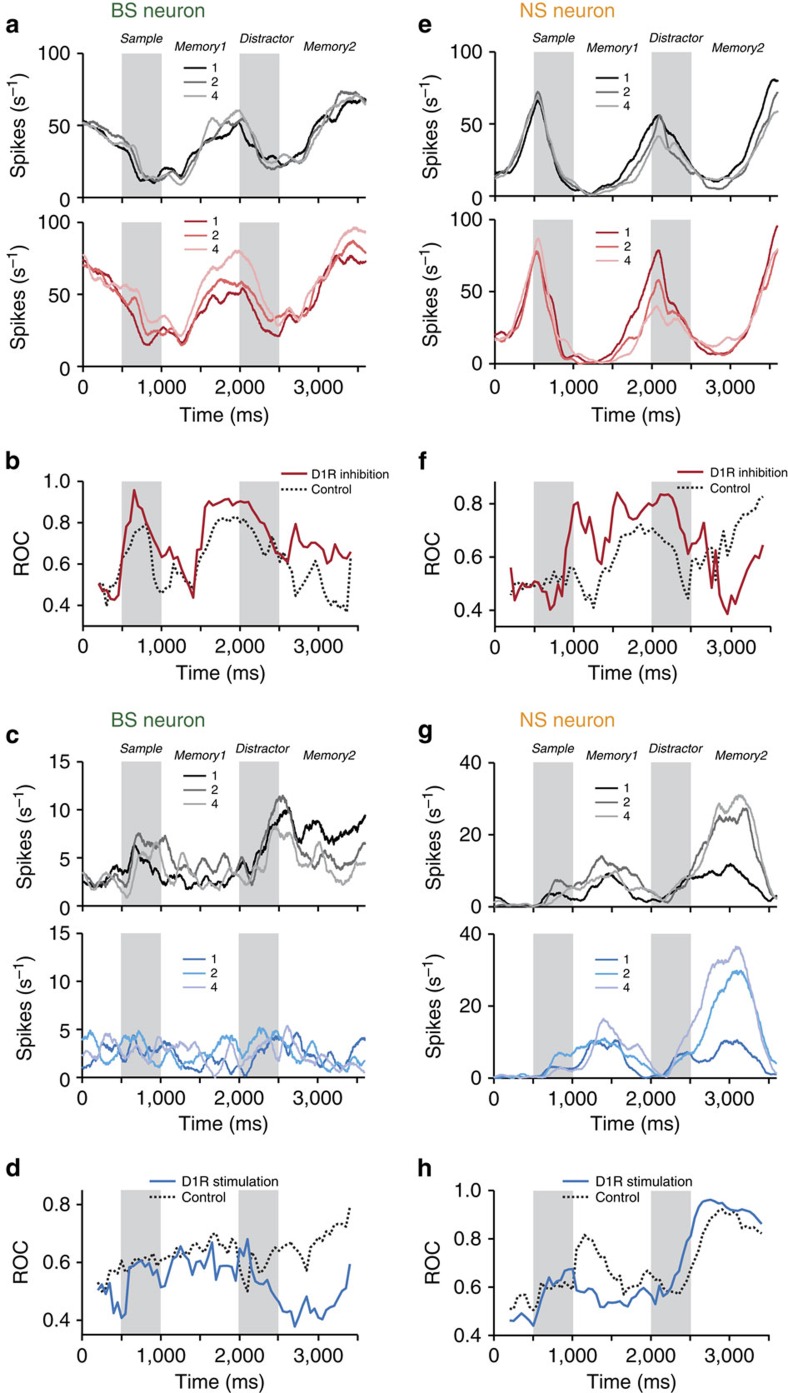
Sample coding in example single neurons. (**a**) Top: spike density histograms for an example sample-selective BS neuron recorded in control trials. Trials are sorted by sample. Bottom: same neuron recorded during D1R inhibition (SCH23390). (**b**) Sliding window ROC analysis quantifying sample numerosity selectivity in control and SCH23390 trials for the neuron in **a**. (**c**,**d**) Same layout as in **a** and **b** for a different sample-selective BS neuron in control trials and with D1R stimulation (SKF38393). (**e**,**f**) Same layout as in **a** and **b** for a sample-selective NS neuron in control trials and with D1R inhibition (SCH23390). (**g**,**h**) Same layout as in **e** and **f** for a different sample-selective NS neuron in control trials and with D1R stimulation (SKF38393).

An example sample-selective BS neuron was tuned to numerosity in several trial epochs under control conditions (top panel, Fig. 3a). Firing rates for the individual numerosities diverged more after blocking D1Rs with SCH23390, indicating that sample-coding strength had improved (bottom panel, Fig. 3a). To quantify the sample-coding strength in control and drug trials, we calculated the time-resolved area under the receiver operating characteristic (ROC) using firing rates for the neuron's preferred and non-preferred numerosity (defined by the highest and lowest firing rate, respectively, on a per-bin basis; that is the ROC measures the degree of momentary sample selectivity; Fig. 3b). Values of 0.5 indicate no separation, values of 1 signal perfect discriminability. For the example BS neuron, target ROC values after D1R inhibition were higher compared with the control condition in all trial epochs, including in the second memory period after the interfering stimulus (Fig. 3b).

A different BS neuron was selective for the sample in particular in the second memory period in control trials (top panel, Fig. 3c). When the D1R agonist SKF38393 was applied, sample selectivity was lost almost entirely (bottom panel, Fig. 3c). The ROC analysis confirmed this reduction, demonstrating the opposite effect on target-coding strength compared to D1R inhibition (Fig. 3d).

In a typical sample-selective NS neuron, we observed the same SCH23390-induced enhancement of target stimulus coding as in BS neurons in the sample and first memory epochs (Fig. 3e,f). However, after presentation of the distractor, sample coding was reduced, not strengthened, by blocking D1Rs. This differential, temporally specific effect of D1Rs on sample selectivity was confirmed by experiments with D1R stimulation. Iontophoretic application of SKF38393 to a different NS neuron enhanced tuning after presentation of the distractor, but showed a tendency to reduce sample coding in the first half of the trial (Fig. 3g,h). Thus, as in BS neurons, D1R inhibition and D1R stimulation had opposing effects on the neuronal representation of target stimuli.

### Differential modulation of target stimuli by D1Rs

**Figure 4:**
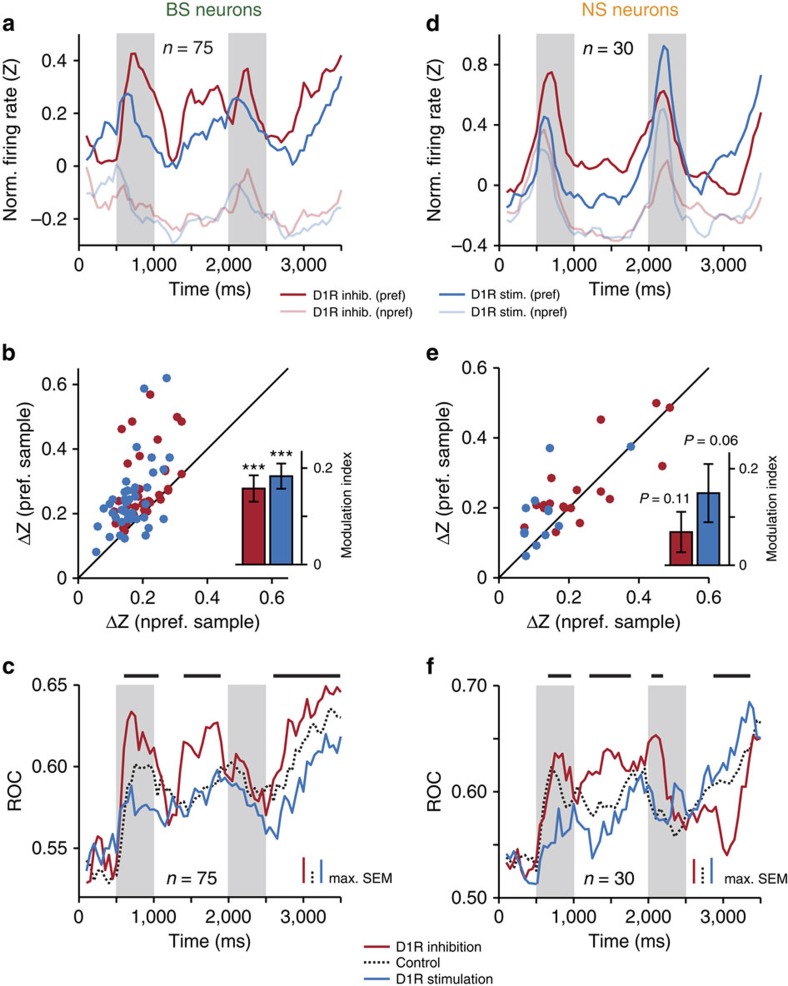
D1R modulation of neuronal sample selectivity. (**a**) Mean normalized firing rates across sample-selective BS neurons (*P*<0.01 in any task epoch) that were recorded either with D1R inhibition (SCH23390, *n*=33) or with D1R stimulation (SKF38393, *n*=42). Data are presented for preferred and non-preferred sample numerosities in SCH23390 and SKF38393 trials. (**b**) Absolute difference in normalized firing rates compared with control conditions for preferred and non-preferred sample numerosities shown for each neuron in **a**. Inset: modulation indices quantifying the degree to which D1R inhibition or D1R stimulation modified firing rates either for the preferred stimulus (positive values) or the non-preferred stimulus (negative values). (**c**) Sliding window receiver operating characteristic (ROC) analysis quantifying the discriminability of the preferred versus non-preferred sample numerosity, that is, sample-coding strength, for the BS neurons in **a** and **b** in control conditions, with D1R inhibition and with D1R stimulation (mean across neurons). Horizontal bars above the curves denote the time bins where sample-coding strength was significantly modulated by the level of D1R activation (Kruskal–Wallis test, *P*<0.01). (**d**–**f**) Same conventions as in **a**–**c** for sample-selective NS neurons (*P*<0.01 in any task epoch) that were recorded either with D1R inhibition (SCH23390, *n*=18) or with D1R stimulation (SKF38393, *n*=12). Error bars, s.e.m. across neurons; ***P<0.001.

To determine in more detail which aspect of neuronal coding of the task-relevant stimulus was modulated by D1Rs, we compared the mean normalized responses to the preferred and non-preferred sample (calculated per bin). In the population of sample-selective BS neurons, D1R inhibition (*n*=33) and D1R stimulation (*n*=42) influenced the neurons' responses to preferred target numerosities, but had little effect on the representation of non-preferred stimuli (Fig. 4a). The firing rate difference between drug trials and control trials was larger for preferred than for non-preferred samples in the vast majority of BS single neurons recorded with either D1R inhibition or D1R stimulation (Fig. 4b). We calculated a modulation index for both drugs that confirmed preferential regulation of preferred stimuli (indicated by positive values, see Methods; Wilcoxon signed-rank test against modulation index of 0; Fig. 4b, inset).

The changes in the separation of preferred stimuli from non-preferred stimuli resulted in drug-induced differences in coding strength in the population of BS neurons. As observed in single BS neurons (Fig. 3a–d), ROC values increased after SCH23390 application, and decreased after SKF38393 application compared with control conditions (Fig. 4c). We found significant differences in the sample, first memory and, importantly, in the second memory period before the behavioural response (bin-wise Kruskal–Wallis tests, *P*<0.01).

Comparable effects of D1R modulation on the representation of the preferred numerosity in NS neurons were seen in the sample and first memory epochs: while the mean responses to non-preferred stimuli were almost indistinguishable for SCH23390 (*n*=18) and SKF38393 application (*n*=12), the responses to the preferred stimuli were clearly different (Fig. 4d). This pattern was less coherent in the second half of the trial following the distractor. Thus, when we calculated the drug-induced change in firing rates compared with control trials for preferred and for non-preferred stimuli across all trial epochs as in BS neurons, the modulation indices did not reach significance for either drug (Fig. 4e). More specifically, the modulation indices were significantly positive during encoding and memorizing of the target stimulus (sample and first memory epoch; SCH22390: 0.08±0.05, *P*=0.05; SKF38393: 0.17±0.06, *P*=0.02), but not during its recovery (distractor and second memory period: SCH22390: 0.04±0.06, *P*=0.68; SKF38393: 0.13±0.08, *P*=0.15). In contrast, modulation indices for BS neurons were consistently positive in both the first half (SCH22390: 0.22±0.04, *P*<0.001; SKF38393: 0.16±0.03, *P*<0.001) and second half of the trial (SCH22390: 0.09±0.04, *P*=0.02; SKF38393: 0.20±0.03, *P*<0.001).

Sample-coding strength across NS neurons, determined by ROC analysis, was amplified by D1R inhibition and reduced by D1R stimulation compared with the control condition in the first half of the trial, as in BS neurons (Fig. 4f). However, the effect of D1R manipulation was reversed in the second memory epoch: increasing D1R activity, not decreasing as in BS neurons, strengthened the recovery of sample information after presentation of the distractor. This result was predicted by the analysis of firing rates in NS neurons (Fig. 4d): in SCH23390 trials, the separation between preferred and non-preferred numerosities reached a minimum in the second memory epoch, while it peaked after application of SKF38393.

In summary, these results suggest that prefrontal D1Rs are able to modulate the level of task-relevant information carried by both BS and NS neurons throughout the trial, in particular after presentation of the task-irrelevant interfering stimulus. Increasing D1R activity here enhanced (putative interneurons) or attenuated (putative pyramidal neurons) the recovery of target information required to complete the task.

### D1R modulation of distractor coding strength

A large proportion of sample-selective neurons also encoded the distractor (35/75 BS neurons, 47%; 25/30 NS neurons, 83%; three-way ANOVA as above, evaluated at *P*<0.01 in the distractor and second memory epoch; note that the number of task epochs during which a neuron might become selective for the distractor was half that for the sample). The difference between distractor-selective BS and NS neurons was significant (*P*<0.001, Fisher's exact test). To determine whether distractor information is also modulated by D1R, we analysed the effects of SCH23390 and SKF38393 on the responses to the task-irrelevant stimulus in the same population of neurons as above. Comparable results were obtained when the analysis was performed for all distractor-selective neurons, irrespective of whether they encoded the sample or not (BS: *n*=52, NS: *n*=26; three-way ANOVA, *P*<0.01).

**Figure 5:**
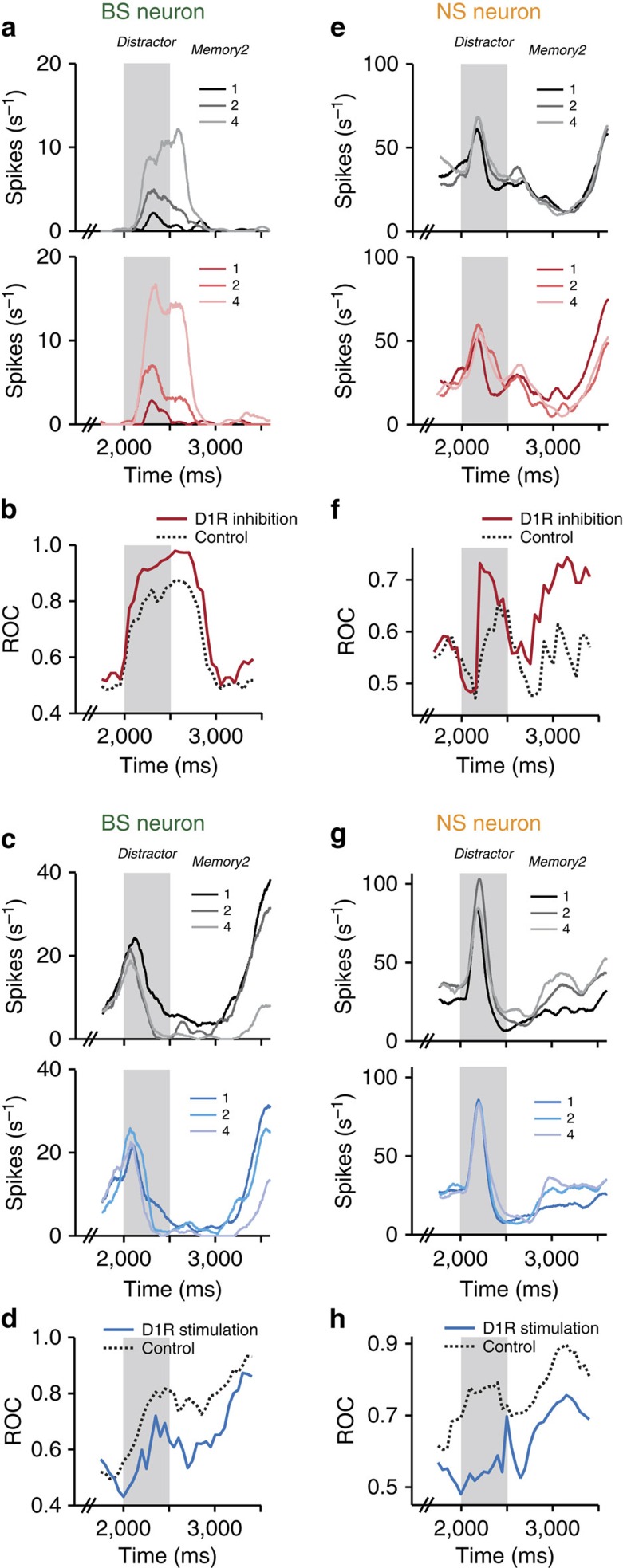
Distractor coding in example single neurons. (**a**) Top: spike density histograms for an example BS neuron recorded in control trials. Trials are sorted by distractor. Bottom: same neuron recorded during D1R inhibition (SCH23390). (**b**) Sliding window ROC analysis quantifying distractor numerosity selectivity in control and SCH23390 trials for the neuron in **a**. (**c**,**d**) Same layout as in **a** and **b** for a different BS neuron in control trials and with D1R stimulation (SKF38393). (**e**,**f**) Same layout as in **a** and **b** for a NS neuron in control trials and with D1R inhibition (SCH23390). (**g**,**h**) Same layout as in **e** and **f** for a different NS neuron in control trials and with D1R stimulation (SKF38393).

An example BS neuron showed strong selectivity for the distractor in the distractor epoch and at the beginning of the subsequent memory epoch under control conditions (top panel, Fig. 5a). After blocking D1Rs with SCH23390, the neuron's firing rates for the individual numerosities separated even more (bottom panel, Fig. 5a), leading to an increase in distractor information carried by this single unit (Fig. 5b). In a different BS neuron, tuning to the distractor numerosity was particularly prominent preceding the test stimulus, but decreased after application of the D1R agonist SKF38393 (Fig. 5c). Stimulation of D1Rs uniformly reduced distractor coding strength throughout the trial in this neuron (Fig. 5d). The same pattern of distractor strengthening by D1R inhibition and distractor attenuation by D1R stimulation was observed in individual NS neurons (Fig. 5e,f and Fig. 5g,h, respectively).

**Figure 6:**
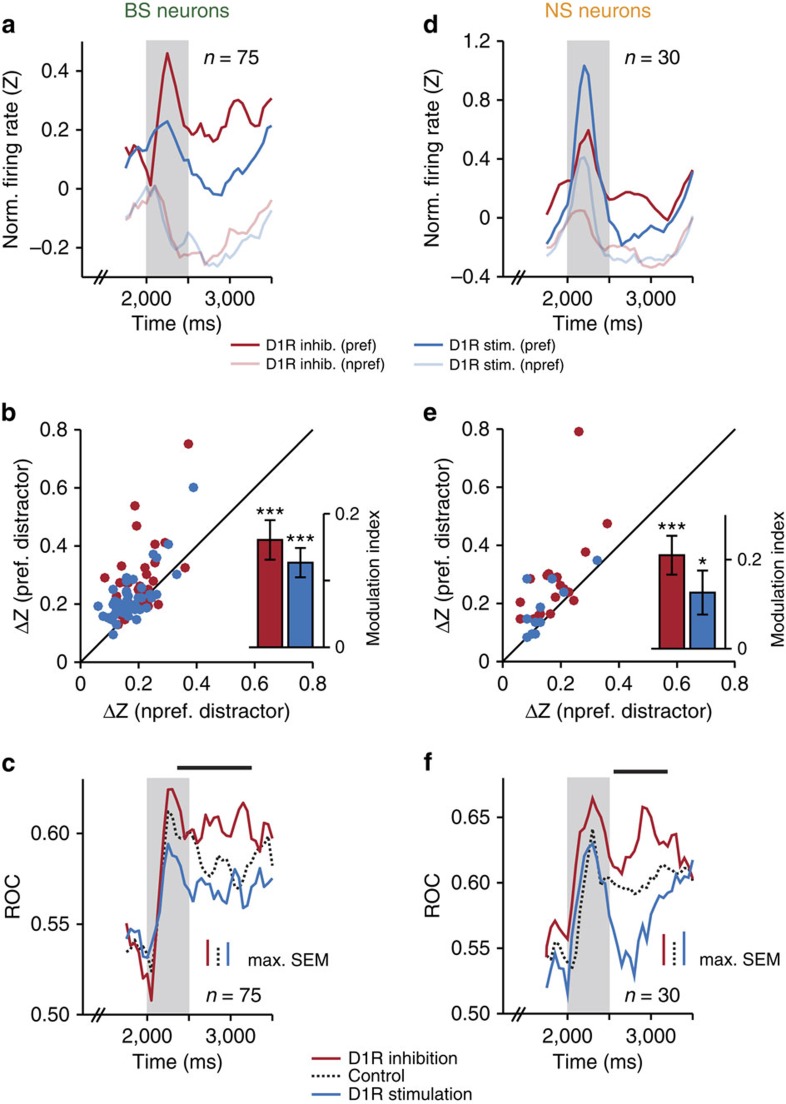
D1R modulation of neuronal distractor selectivity. (**a**) Mean normalized firing rates across BS neurons (same neurons as in Fig. 4) that were recorded either with D1R inhibition (SCH23390, *n*=33) or with D1R stimulation (SKF38393, *n*=42). Data are presented for preferred and non-preferred distractor numerosities in SCH23390 and SKF38393 trials. (**b**) Absolute difference in normalized firing rates compared with control conditions for preferred and non-preferred distractor numerosities shown for each neuron in **a**. Inset: modulation indices quantifying the degree to which D1R inhibition or D1R stimulation modified firing rates either for the preferred stimulus (positive values) or the non-preferred stimulus (negative values). (**c**) Sliding window receiver operating characteristic (ROC) analysis quantifying the discriminability of the preferred versus non-preferred distractor numerosity, that is, distractor coding strength, for the BS neurons in **a** and **b** in control conditions, with D1R inhibition and with D1R stimulation (mean across neurons). Horizontal bars above the curves denote the time bins where distractor coding strength was significantly modulated by the level of D1R activation (Kruskal–Wallis test, *P*<0.01). (**d**–**f**) Same conventions as in **a**–**c** for NS neurons (same neurons as in Fig. 4) that were recorded either with D1R inhibition (SCH23390, *n*=18) or with D1R stimulation (SKF38393, *n*=12). Error bars, s.e.m. across neurons; *P<0.05; ***P<0.001.

We found that these single-cell responses were typical for the entire population of analysed neurons (Fig. 6). As seen for the encoding of the task-relevant sample, changes in the level of prefrontal D1R activity affected the representation of the preferred distractor numerosity in BS neurons, not the non-preferred distractor (Fig. 6a). Modulation indices were significantly positive for both D1R inhibition with SCH23390 and D1R stimulation with SKF38393 (Wilcoxon signed-rank tests; Fig. 6b). Distractor coding strength across all analysed BS neurons improved after blocking D1Rs and reduced after stimulating D1Rs (bin-wise Kruskal–Wallis tests, *P*<0.01; Fig. 6c).

The interfering distractor was modulated in the same manner in the group of NS neurons. The separation between the preferred and non-preferred distractor was high in the second memory epoch after SCH23390 application, but was reduced to a minimum after D1R stimulation with SKF38393 (Fig. 6d). Modulation indices were significantly positive for both drugs (Fig. 6e). Overall, D1R inhibition enhanced the representation of the distractor in NS neurons, whereas it was attenuated by D1R stimulation (Fig. 6f). These results indicate that D1Rs control not only the level of task-relevant information in local PFC circuits, but also determine how strongly irrelevant interfering stimuli are encoded.

### D1R modulation of behaviour

**Figure 7:**
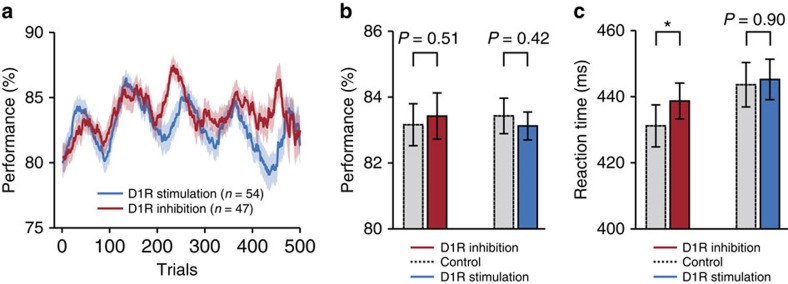
Drug effects on behavioural performance. (**a**) Behavioural performance (per cent correct trials) averaged across sessions where control blocks alternated with D1R inhibition (SCH23390; *n*=47) and where control blocks alternated with D1R stimulation (SKF38393; *n*=54). Data are presented as a moving average. (**b**) Mean behavioural performance in control and drug trials shown separately for sessions with D1R inhibition (left) and D1R stimulation (right). (**c**) Mean reaction times (correct match trials) in control and drug trials shown separately for sessions with D1R inhibition (left) and D1R stimulation (right). Error bands and bars, s.e.m. across sessions; *P<0.05.

Finally, we asked whether the neuronal changes by D1R modulation resulted in a shift in the monkeys' behavioural responses and their ability to resist the distracting stimuli. Iontophoretic drug application is highly focal^32^, and most primate studies that iontophoretically applied drugs to the cortex did not report any behavioural changes^19^,^20^,^33^,^34^,^35^. We found that the monkeys' performance oscillated over time both in sessions where SCH23390 was applied and in sessions where SKF38393 was used (Fig. 7a). The cycle of this oscillation closely matched the length of the control and drug blocks in our study (∼100 trials), making it difficult to dissociate pharmacological effects on the animals' behaviour from non-specific effects of for example motivation and arousal. We controlled for slow trends in behavioural performance (‘warming up effect' at the beginning of each session, Fig. 7a) by removal of the first control block before analysis. There was no significant difference between control and drug trials in either the SCH23390 or SKF38393 sessions (Fig. 7b, Wilcoxon signed-rank tests). Reaction times (correct match trials) were increased in blocks with D1R inhibition compared with control trials, but there was no corresponding decrease in blocks with D1R stimulation (Fig. 7c, Wilcoxon signed-rank tests). Thus, as expected, the focal microiontophoretic drug application did not yield a systematic change in behavioural performance.

## Discussion

Dopamine modulates a broad range of prefrontal cognitive functions in the primate^10^. D1Rs are of particular importance for working memory performance. It has been firmly established that D1Rs control the ability to memorize, for example, a location in space in monkeys^17^,^18^ and humans^36^,^37^. We now show that D1Rs modulate the extent to which task-relevant information is recovered in prefrontal working memory circuits following interference by a distracting stimulus. The direction of modulation depended on the encoding cell type. In putative pyramidal neurons, D1R inhibition enhanced and D1R stimulation reduced the representation of the target stimulus before the animals' behavioural choice. The opposite pattern was observed in putative interneurons.

Both drugs were ejected with currents of the same polarity, yet consistently produced opposing effects on neuronal coding compared with the control condition (Figs 3, 4, 5, 6). This cannot be explained by the action of iontophoretic current, but instead shows that the representation of targets and distractors in PFC was modulated specifically by D1Rs.

Reducing the level of D1R activity in BS neurons increased sample-coding strength throughout the trial (Figs 3 and 4). Sample information was modulated in the same direction both at the beginning of the trial, when only one stimulus had to be memorized, and at the end, when the distractor occupied working memory resources (Figs 5 and 6) and the sample had to be recovered. Thus, the D1R-dependent coding scheme for the target stimulus in BS neurons was comparable under two distinct conditions, offering a robust and straightforward decoding option for task-relevant information.

In contrast, the direction of modulation of task-relevant information in NS neurons changed after presentation of the distractor (Figs 3 and 4). The two major classes of cortical neurons, interneurons and pyramidal cells, assume complementary roles in many prefrontal cognitive tasks such as working memory^28^, categorization^31^,^38^,^39^, sensory processing and perceptual decision making^24^,^38^,^40^ and attentional control^41^. Here we also observed cell-type-specific differences in the way sample and distractor information were encoded after memory interference. The switch in D1R modulation together with the finding that distractor information was present in more NS than BS neurons suggest that distinct D1R-dependent physiological mechanisms underlie the retrieval of behaviourally relevant memories in the two cell classes.

Distracting stimuli were also subject to modulation by prefrontal D1Rs in both cell types (Figs 5 and 6). This finding is in line with the observation that the neuronal responses to the distractor were not actively suppressed, and that the same neurons carry information about both the task-relevant and the interfering stimulus (compare sample and distractor coding strength in Figs 4c,f and 6c,f). Thus, correctly selecting the target, not filtering the distractor, in the second memory period is required to solve the present task^7^. The modulation of task-relevant information by D1Rs could therefore represent an adequate and effective mechanism to influence behavioural outcome, although we were not able to detect robust changes in the animals' performance due to the highly focal pharmacological manipulation of dopamine receptors (Fig. 7).

It is important to note that the modulation of both sample and distractor information in the second half of the trial is not a trivial finding. This is because our balanced task design (Methods) ensures that a change in neuronal activity that only depends on the numerosity of either one of the stimuli would factor out when trials are sorted by the other stimulus. Instead, dual modulation before the animals' behavioural choice indicates that neuronal firing rates in this trial epoch are determined by a conjunction of activity, reflecting the maintenance of both the sample and distractor numerosity in working memory.

Midbrain dopamine neurons fire phasic bursts in response to behaviourally relevant sensory events^12^,^42^,^43^. It has been suggested that prefrontal dopamine could serve to modulate a signal's saliency^24^,^44^. In support of this idea, D1Rs have been shown to regulate a variety of neuronal representations in PFC, such as visual shapes of different complexity^15^,^24^, spatial locations^19^,^20^ and abstract rules^16^. Several studies have observed enhanced coding after application of D1R agonists and attenuated responses for D1R antagonists^15^,^16^,^20^, while others have found the opposite^14^,^19^. In accordance with the latter reports, we found that blocking D1Rs predominantly strengthened task-relevant information and that stimulating D1Rs reversed this effect (Figs 4c,f and 6c,f). The reasons for these diverging findings across studies are not yet clear. Our results show that the neuronal cell type is an important factor that has to be taken into account^24^. The modulatory effects of dopaminergic drugs in PFC also vary depending on concentration, with the dose–response curve assuming an inverted U-shape^20^. However, the ejection currents in the present study were chosen to match the most effective and commonly used dosages reported by others and are therefore unlikely to be solely responsible for the observed response profiles. It is further conceivable that the manner in which the dopamine system controls neuronal processing is influenced by and adapted to the individual task demands and context (and thus the necessary processing stages). In support of this notion, the lack of suppression of distractor responses in this study and our previous experiments^7^ sets the current task apart from other protocols that also investigated the coding of task-irrelevant distractors in PFC, but using different stimuli^45^,^46^.

While D1Rs represent the dominant dopamine receptor family in PFC and are expressed by about one-quarter of all prefrontal neurons, the D2R family is concentrated in layer V and has therefore mainly been associated with motor output signals^14^,^47^,^48^. Additional studies are required to determine the role of prefrontal D2Rs in controlling working memory circuits.

Our results support an active role of prefrontal D1Rs, beyond passive stimulus maintenance, at the center of cognitive control operations. It is tempting to speculate that unregulated dopamine signals could constitute an important factor in the generation of mental disorders. For example, one of the most influential theories to explain the psychotic symptoms in schizophrenia is the concept of aberrant salience^23^,^49^, whereby an excess of dopamine could make it impossible to resist irrelevant and interfering sensory input, resulting in hallucinations and intrusions of thought. Potential neurobiological mechanisms have been lacking. By adaptable and context-dependent modulation of prefrontal processing, dopamine could assume a central role in higher cognitive functioning in health and disease.

## Methods

### Surgical procedures

Two adult male rhesus monkeys (*Macaca mulatta*, monkey R and monkey W) were implanted with a titanium head post and a right-hemispheric recording chamber centered over the principal sulcus of the lateral prefrontal cortex (PFC), anterior to the frontal eye fields, guided by anatomical MRI and stereotaxic measurements. Chambers were angled to be able to penetrate the cortical surface perpendicularly. Surgery was conducted using aseptic techniques under general anaesthesia. All experimental procedures were in accordance with the guidelines for animal experimentation approved by the responsible authority, the Regierungspräsidium Tübingen.

### Task and stimuli

The monkeys were trained to match visually presented non-symbolic set sizes (numerosities) while suppressing a salient task-irrelevant, interfering numerosity (Fig. 1)^7^. The animals grabbed a bar to initiate a trial and maintained eye fixation within 1.75° of visual angle of a central white dot. An infrared-based eye tracking system monitored ocular position (ISCAN). Trials were immediately aborted and excluded from further analysis if the animals broke fixation. Stimuli were presented on a centrally placed grey circular background subtending 5.4° of visual angle. Following a 500 ms pre-sample (pure fixation) period, a 500 ms sample stimulus containing 1, 2 or 4 dots was shown. The monkeys had to memorize the sample numerosity for 2,500 ms and compare it with the number of dots (1, 2 or 4) presented in a 1,000 ms test stimulus. Test stimuli were marked by a red ring surrounding the background circle. Thus, there were virtually no early release errors in both animals (monkey R:<0.01%; monkey W:<0.2%), that is, they did not confuse the test with the interfering stimulus. If the numerosities matched (50% of trials), the animals released the bar (correct Match trial). If the numerosities were different (50% of trials), the animals continued to hold the bar until the matching number was presented in the subsequent image (correct Non-match trial). Match and Non-match trials were pseudorandomly intermixed. Correct trials were rewarded with a drop of water. In 75% of trials, a 500 ms interfering numerosity (1, 2 or 4 dots) was presented between the sample and test stimulus. The interfering numerosity was not systematically related to either the sample or test numerosity and therefore not required to solve the task. In 25% of trials, a 500 ms grey background circle without dots was presented instead of an interfering stimulus, that is, trial length remained constant (control condition, blank). Trials with and without interfering numerosities were pseudorandomly intermixed. Stimulus presentation was balanced. That is, across a set of trials in which a given numerosity was used as the sample, it was followed by all interfering numerosities with equal frequency. Similarly, a given interfering numerosity was preceded by all sample numerosities with equal probability. Thus, when trials are sorted by sample (or interfering numerosity), any measure of neuronal activity is directly attributable to that stimulus, because the influence of the interfering numerosity (or sample) is factored out across trials.

Low-level, non-numerical visual features could not systematically influence task performance^26^: in half of the trials, dot diameters were selected at random. In the other half, dot density and total occupied area were equated across stimuli. CORTEX software (NIMH) was used for experimental control and behavioural data acquisition. All stimuli were produced using MATLAB (The Mathworks) and generated anew before every recording session to ensure that the animals could not solve the task by memorizing stimulus sequences.

### Extracellular recordings and iontophoretic drug application

In each recording session, up to three custom-made tungsten-in-glass electrodes flanked by two pipettes each^24^,^50^ were inserted transdurally using a modified electrical microdrive (NAN Instruments, Israel). Single neurons were recorded at random; no attempt was made to preselect the neurons for task-related activity or based on drug effects. Signal acquisition, filtering, amplification and digitalization were accomplished with the MAP system (Plexon). Waveform separation was performed offline (Offline Sorter; Plexon). Drugs were applied iontophoretically (MVCS iontophoresis system; npi electronic, Germany). Electrode impedance and pipette resistance were measured after each recording session. Electrode impedances were 0.6–3 MΩ (measured at 500 Hz; Omega Tip Z; World Precision Instruments). Pipette resistances depended on the pipette opening diameter, drug and solvent used. Typical resistances were 15–50 MΩ (full range, 10–180 MΩ). Retention currents of –7 nA were used to hold the drug in the pipette during control conditions. The ejection current for SCH23390 (10 mM in double-distilled water, pH 4.0 with HCl; Sigma-Aldrich) was +25 nA. The ejection current for SKF38393 (10 mM in double-distilled water, pH 4.0 with HCl; Sigma-Aldrich) was +15 nA. One pipette per electrode was filled with drug solution, and the other always contained 0.9% NaCl to prevent this capillary from taking up any excess drug solution during filling. In each recording session, control conditions using the retention current alternated with drug conditions using the ejection current. Drugs were applied continuously for 12–15 min (drug conditions), depending on the number of trials completed correctly by the animal. Each control or drug application block consisted of 50–100 correct trials to yield sufficient data for analysis. The first block was always the control condition. We analysed trials if the recorded currents were in the range of −7±2 nA (control), +25±5 nA (SCH23390) or +15±5 nA (SKF38393). All other trials were excluded. To avoid contaminating data at the beginning of each block, we also discarded any trials that followed within 1 minute of a block switch (from control to drug or vice versa).

### Data analysis

Data analysis was performed with MATLAB. None of the reported analyses depended on the exact choice of trials to include or time windows to analyse. Repeating analyses with a different set of parameters yielded comparable results.

*Sample-selective neurons*. Neurons were included in the analysis if the following criteria were met: first, their average firing rate across trials was at least 1 spike per s; and second, they were recorded for at least 1 correct trial in all 24 conditions (3 sample numerosities × 4 interfering numerosities including the blank [0] stimulus × 2 iontophoresis conditions (control or drug)). A total of *n*=246 neurons fulfilled these criteria.

A three-way ANOVA was calculated with main factors sample numerosity (1, 2 or 4), interfering numerosity (1, 2 or 4) and iontophoresis condition (control or drug) using average firing rates in the sample, first memory, distractor and second memory periods, including correct trials only. Neurons with a significant main effect of sample numerosity in any trial period (*P*<0.01; *n*=122) were classified as sample selective.

*Single-cell and population responses*. For single-cell spike density histograms, the average firing rate across trials sorted by sample or interfering numerosity (correct trials only) was smoothed with a Gaussian kernel (bin width of 150 ms, steps of 1 ms). For the population responses, we first normalized (*Z*-scored) the responses of a given unit by subtracting the mean response from its firing rate and by dividing the result by the s.d. of the responses. Both the mean and the s.d. were computed by combining the unit's responses across all trials and time bins (width of 200 ms, steps of 50 ms) using correct trials only. We then defined a neuron's preferred and non-preferred numerosity by its maximum and minimum firing rate, respectively, for every bin individually and averaged normalized firing rates across neurons.

*ROC analysis*. Neuronal coding strength (selectivity) was quantified using receiver operating characteristic (ROC) analysis^51^. The area under the ROC curve is a non-parametric measure of the discriminability of two distributions. It denotes the probability with which an ideal observer can tell apart a meaningful signal from a noisy background. Values of 0.5 indicate no separation, values of 1 signal perfect discriminability. The ROC takes into account both the difference between distribution means as well as their widths and is therefore a suitable indicator of signal quality. We calculated the ROC for each neuron using the firing rate distributions of the preferred and the non-preferred numerosity (determined for every bin individually as described above). Sliding ROC analysis was performed with overlapping 200 ms windows stepped in 50 ms increments. Population ROC values in control and drug trials were compared using bin-wise Kruskal–Wallis tests (*P*<0.01 after correcting for multiple comparisons by dividing the original significance threshold by the number of comparisons made across each window).

*Drug modulation index*. The modulation index (MI) describes whether SCH23390 or SKF38393 application had a greater effect on the firing rates for the preferred (MI>0) or non-preferred numerosity (MI<0). For all analysed neurons, we calculated the average absolute difference in *Z*-scores between drug and control trials for the preferred and non-preferred stimulus (Δ*Z*_pref_ and Δ*Z*_npref_, respectively). We used absolute values to account for reversals in the direction of firing rate modulation in the course of the trial (Fig. 4d). The MI was then defined as: MI=(Δ*Z*_pref_–Δ*Z*_npref_)/(Δ*Z*_pref_+Δ*Z*_npref_).

*Extracellular waveforms*. Recorded single units were categorized into narrow-spiking (NS) and broad-spiking (BS) neurons, that is, putative interneurons and pyramidal cells, using a linear classifier (k-means, *k*=2, squared Euclidean distance)^24^,^31^. For each single unit, the template waveform was extracted with the Offline Sorter (Plexon). Only neurons with a downward voltage deflection followed by an upward peak were included. Units with a minimum outside 200–400 μs or a maximum before 300 μs after reaching the initial threshold were excluded. Waveforms were normalized by their difference between maximum and minimum voltage deflection and aligned to their minimum. Units in the cluster with the smaller mean spike width constituted the population of narrow-spiking neurons, and units in the cluster with the larger mean spike width constituted the broad-spiking neurons.

*Behavioural data*. Only sessions with at least 25 correct control and 25 correct drug trials were analysed. The moving average of behavioural performance (per cent correct trials) was calculated for SCH23390 and SKF38393 sessions (*n*=47 and *n*=54, respectively) using a window size of 40 trials. We controlled for slow trends in behavioural performance (‘warming up effect' at the beginning of each session, Fig. 7a) by removal of the first control block prior to statistical comparisons. Reaction times (RT) were determined for correct match trials only (in non-match trials, the second test image following the non-match was always a match and therefore predictable). Session averages for control and drug conditions were compared using Wilcoxon signed-rank tests (paired data).

### Data availability

The data that support the findings of this study are available from the corresponding author on request.

## Additional information

**How to cite this article**: Jacob, S. N. *et al*. Cell-type-specific modulation of targets and distractors by dopamine D1 receptors in primate prefrontal cortex. *Nat. Commun.* **7**, 13218 doi: 10.1038/ncomms13218 (2016).
